# RETRACTION: Analysis of circRNA‐mRNA Expression Profiles and Functional Enrichment in Diabetes Mellitus Based on High Throughput Sequencing

**DOI:** 10.1111/iwj.70556

**Published:** 2025-04-18

## Abstract

**RETRACTION:** ZhaoW. , MengX. , and LiangJ. , “Analysis of circRNA‐mRNA Expression Profiles and Functional Enrichment in Diabetes Mellitus Based on High Throughput Sequencing,” International Wound Journal 20, no. 5 (2023): 1253–1262, 10.1111/iwj.13838.35504843 PMC9284653

The above article, published online on 03 May 2022, in Wiley Online Library (http://onlinelibrary.wiley.com/), has been retracted by agreement between the journal Editor in Chief, Professor Keith Harding; and John Wiley & Sons Ltd. Following an investigation by the publisher, all parties have concluded that this article was accepted solely on the basis of a compromised peer review process. In addition, further investigation by the publisher found discrepancies between the figures and data and the statements made in the results, contradictions between the methodology and data, and flaws in the research concept and design that fundamentally compromise the validity of the results presented in the article. The editors have therefore decided to retract the article. The authors did not respond to our notice regarding the retraction.

**RETRACTION:** W. Zhao , X. Meng , and J. Liang , “Analysis of circRNA‐mRNA Expression Profiles and Functional Enrichment in Diabetes Mellitus Based on High Throughput Sequencing,” International Wound Journal 20, no. 5 (2023): 1253–1262, 10.1111/iwj.13838.35504843 PMC9284653

The above article, published online on 03 May 2022, in Wiley Online Library (http://onlinelibrary.wiley.com/), has been retracted by agreement between the journal Editor in Chief, Professor Keith Harding; and John Wiley & Sons Ltd. Following an investigation by the publisher, all parties have concluded that this article was accepted solely on the basis of a compromised peer review process. In addition, further investigation by the publisher found discrepancies between the figures and data and the statements made in the results, contradictions between the methodology and data, and flaws in the research concept and design that fundamentally compromise the validity of the results presented in the article. The editors have therefore decided to retract the article. The authors did not respond to our notice regarding the retraction.

